# Supplementary material for: Complexome profiling of the Chlamydomonas psb28 mutant reveals TEF5 as an early PSII assembly factor
Source: Plant Cell. 2025 Mar 17;37(6):koaf055. doi: 10.1093/plcell/koaf055 (PMC12164590; doi:10.1093/plcell/koaf055)
Supplement: koaf055_Supplementary_Data [file koaf055_supplementary_data.zip › psb28_interactive.zip!/plots/intensity/qualityPlots/peptides/TRXH2_Cre09.g391900.t1.1.html]

### Cre09.g391900.t1.1

Trivial name: TRXH2  
SeqCoverage [median]: 20.40  
Mass: 11.84 kDa  
  
 

| Sequence | MissedCleavage | Mass [Da] | wtIntensity | muIntensity | Score |
| --- | --- | --- | --- | --- | --- |
| ADDLVGASQDKLK | 1 | 1358.70 | 1241.35 | 8887.33 | 84.29 |
| GGSVIVIDSK | 0 | 973.54 | 2617.79 | 6602.84 | 93.65 |
|  |  |  | 3859.1 | 15490.2 |  |
